# Tackling HCV-3 in Asia: Breakthroughs for Efficient and Cost-effective Treatment Strategies

**DOI:** 10.5005/jp-journals-10018-1163

**Published:** 2016-07-09

**Authors:** Naba Saeed, Ahmet Gurakar

**Affiliations:** 1 Department of Transplant Hepatology, Division of Gastroenterology and Hepatology, Johns Hopkins University School of Medicine, Baltimore, Maryland, USA; 2 Department of Transplant Hepatology, Johns Hopkins University School of Medicine, Division of Gastroenterology and Hepatology, Baltimore Maryland, USA

**Keywords:** Genotype 3, Hepatitis C, New therapies, Sofosbuvir.

## Abstract

Hepatitis C virus (HCV) is known to cause chronic hepatitis C, and its sequelae of cirrhosis and hepatocellular carcinoma. Hepatitis C genotype 3 (HCV-3) in particular is notorious for causing accelerated liver fibrosis, cardiovascular, and metabolic effects, thus increasing morbidity and mortality. It is the commonest variant in Asian countries like India and Pakistan. It is also one of the hardest-to-treat genotypes, especially among treatment-experienced and cirrhotic patients. Due to limited health care affordability and accessibility in these areas, many patients remain untreated.

Until recently, the established therapy for HCV had been a combination of pegylated interferon + ribavirin. However, it was only effective in about half of patients and had severe adverse effects; hence a more efficacious option needed to be found. Recent advances have led to the development of sofosbuvir, an NS5B inhibitor that is fast becoming the standard of care, in combination with other novel drugs. It was initially marketed at $1,000 per pill, a cost that was too high for most. Thus, it has not been utilized as a global therapy as yet. Formulation of effective interferon-free regimens is a huge milestone, and awareness needs to be raised regarding these new highly effective options in both the physician and the patient population.

This article discusses the newest drugs and combinations that have been developed in the fight against HCV-3, as a treatment outline for HCV-3-dominant areas. It also highlights recent breakthroughs in cost reductions of these drugs and the effort to make them globally accessible.

**How to cite this article:**

Saeed N, Gurakar A. Tackling HCV-3 in Asia: Breakthroughs for Efficient and Cost-effective Treatment Strategies. Euroasian J Hepato-Gastroenterol 2016;6(1):35-42.

## BACKGROUND

Hepatitis C virus (HCV) is notorious for causing liver cirrhosis and increasing the risk of hepatocellular carcinoma.^1^ In today’s world of innovation, however, it is fast becoming a curable disease. The virus affects approximately 170 million people globally, of which about half are in Asia and the Western Pacific.^2^ It comprises seven genotypes, each with multiple subtypes.^3^ A total of 54.3 million cases are due to HCV genotype 3 (HCV-3), which is the most common variant in many parts of Asia, especially in India, Nepal, and Pakistan.^4^ Other areas where HCV-3 is more prevalent include Australia, Eastern and Western Europe, and Latin America.^4^ Some other genotypes commonly seen in Asia include HCV-1 and 2.^4^

Hepatitis C virus genotype 3 has unique effects on glucose and lipid metabolism,^5^ causing the highest rate of virus-related liver steatosis out of all of the HCV types.^6^ The degree of steatosis correlates directly with HCV-RNA levels; hence eradication of the virus leads to a decrease in steatosis.^7,8^ It also carries a greater risk of cardiovascular disease^9^ and insulin resistance.^10,11^ It is notorious for an accelerated rate of liver fibrosis and an increased mortality compared with HCV-1 and 2, among patients who failed to achieve SVR (sustained virologic response)^12,13^ (Flow Chart 1 outlines these HCV-3-specific characteristics). Due to these alarming characteristics, there is an urgent need for adequate treatment options.

**Flow Chart 1:**
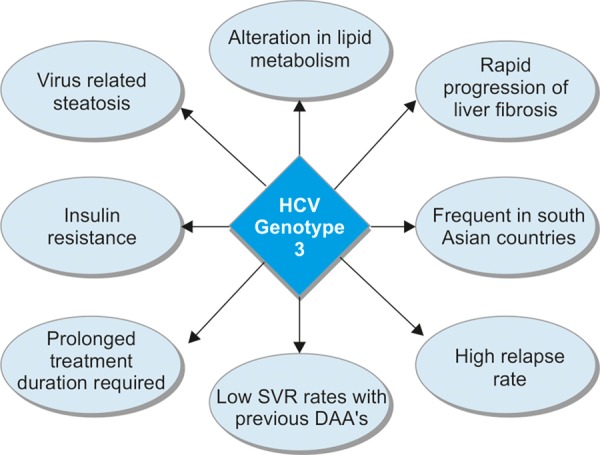
Hepatitis C virus-3 Specific Characteristics^38^

The previously established therapy for HCV-3 thus far had been pegylated interferon (pegIFN) (alpha-2a-180 mg/week or alpha-2b-1.5 mg/kg/week) + ribavirin (RBV) (800–1400 mg/day) for 24 weeks.^14^ However, numerous studies have tried to determine the optimal treatment duration with varying results.^15-17^ Accepted durations included 12 weeks in patients achieving RVR (Rapid Virological Response) (treatment response at 4 weeks), but extending treatment to 36 weeks in those without RVR. Factors that complicate treatment include the presence of cirrhosis, high viral load, no RVR,^18^ and the presence of metabolic abnormalities. The EASL guidelines^19^ took all of these factors into account and thus recommended:

- Treatment for 16 weeks in patients with baseline low viral load (LVL) and RVR;
- At least 24 weeks of therapy in the presence of steatosis, advanced fibrosis, cirrhosis, or metabolic abnormalities like insulin resistance and metabolic syndrome;
- 48 or 72 weeks of treatment in those with early or delayed virologic response, given that HCV-RNA is undetectable at week 24;
- Discontinuation of treatment in those with no RVR, ≤ 2 log_10_ drop in HCV-RNA levels or detectable RNA at week 24.

However, this interferon-based regimen was not without its drawbacks. In patients with HCV-3, SVR was achieved only in 33% of cirrhotic patients, according to Aghemo et al, with a high posttreatment relapse rate.^20^ Furthermore, its use was limited by its wide range of adverse effects, including bone marrow depression, flu-like symptoms, neuropsychiatric disturbances, and autoimmune diseases. Occurrence of these symptoms had decreased the compliance with treatment and the willingness to initiate treatment with interferon.^21^ In some circumstances, use of pegIFN is contraindicated, including decompensated cirrhosis, Model for End Stage Liver Disease score > 10, unstable psychiatric disorders, advanced cardiac or pulmonary disease, autoimmune diseases, and hematological abnormalities.^22^ These problems brought to light the necessity of developing interferon-free regimens and making them available for treatment worldwide, so we could take on the challenge of HCV, especially that of genotype 3.

This article highlights the current breakthroughs in treatment options for HCV-3, focusing on the new NS5B inhibitor sofosbuvir (SOF) and concurrent combinations, and recent advances that have been made in the effort to make these drugs globally accessible and cost-effective.

## CORNERSTONE OF THERAPEUTIC ADVANCES

Sofosbuvir is an HCV-RNA NS5B inhibitor that blocks viral replication.^23^ It is a prodrug that is then converted within hepatocytes to its active form (GS-461203). This active form is then incorporated into HCV-RNA, causing chain termination.^23,24^ GS-461203 does not act on human DNA or RNA.^25^ It is then dephosphorylated to GS-331007, its circulating form, and excreted renally.^23,24^ It was approved by the FDA for use as the first nucleotide therapy against hepatitis C in December 2013 and subsequently was approved by the European Union in January 2014.^26^ Current AASLD guidelines now recommend the use of SOF + RBV for 24 weeks, or an alternative regimen of SOF + pegIFN + RBV for 12 weeks in all genotype 3 patients, as of March 2014.^27^ However, optimal treatment of HCV-3-infected patients is still largely unknown.^28^ Multiple clinical trials have been undertaken to assess the efficacy of different regimens in different patient populations, and to date, regimens with increased SVR rates and decreased side effects and resistance are still evolving.

## IFN-BASED THERAPIES

The Lonestar-2 Trial assessed the use of SOF (400 mg OD) + pegIFN alpha-2a (180 μg once weekly) + RBV (1000 mg/day if < 75 kg or 1200 mg/day if > 75 kg – in two divided doses) for 12 weeks in treatment-experienced (TE) patients with HCV-2 or 3. The primary end point was SVR 12, which was achieved in 83% of genotype 3 patients. The response was the same in cirrhotics and non-cirrhotics (83% in both groups). There were four HCV-3 treatment failures; two patients had virologic relapse, and two were lost to follow-up. However, adverse effects occurred in a substantial number of patients in the study. The conclusion was that SOF + RBV + pegIFN for 12 weeks gave high SVR rates, regardless of cirrhosis.^29^

## IFN-FREE REGIMENS

### Sofosbuvir and Ribavirin

The Electron trial was a phase II trial comparing different combinations of SOF and RBV with or without pegIFN. There were six treatment arms evaluating the optimal therapy for genotypes 2 and 3 in treatment naïve (TN) patients. They all contained combinations of these drugs for 12 weeks, except for arm 5, which tested SOF therapy alone for 12 weeks. All arms showed 100% SVR at week 12, except for SOF alone, which showed only 60% SVR12. The study concluded that SOF + RBV for 12 weeks was an effective treatment.^30^

The Fission trial compared the use of SOF (400 mg OD) + RBV (1000 mg/day if < 75 kg or 1200 mg/day if > 75 kg) for 12 weeks with use of pegIFN alpha-2a (180 μg once weekly) + RBV (fixed dose – 800 mg/day) for 24 weeks in TN chronic HCV-2 and 3 patients. In the SOF + RBV group, 99% patients had achieved SVR by week 4, and remained in remission at the end of treatment. However, SVR at 12 weeks post-therapy had decreased to 67%. Fifty-six percent of these were genotype 3 patients. In the pegIFN/RBV group, 67% patients had achieved SVR at week 4, 89% at the end of therapy, and only 67% remained in remission at week 12 post-therapy. Of these, there were 63% genotype 3 patients with SVR12. Of note, a higher response was seen in cirrhotic patients who were given SOF/RBV (47% with SVR12), in contrast to those given pegIFN/RBV (38% with SVR12). There was a drastic difference in the amount of adverse effects seen between the regimens, with an 11% discontinuation due to adverse effects in the interferon group, but only 1% in the SOF/RBV group. The study determined that SOF + RBV had similar efficacy to pegIFN + RBV, but had fewer side effects.^31^

The Valence study compared the effect of SOF + RBV in TN and TE chronic HCV-2 and 3 patients with placebo. For genotype 3, SOF (400 mg OD) and RBV (1000 mg/day if < 75 kg or 1200 mg/day if > 75 kg) were used for 24 weeks in 250 subjects, compared with 85 patients in the placebo group. SVR12 was 85% for HCV-3; 94% in the TN group and 79% in the TE group. Patients were then further subdivided based on cirrhosis and prior treatment. In TN group, 95% non-cirrhotics and 92% cirrhotics achieved SVR12, whereas in the TE group, 87% non-cirrhotics and only 62% cirrhotics achieved SVR12. No SVR was achieved in the placebo group. Thus, it was concluded that treatment with SOF/RBV for 24 weeks in HCV-3 achieved high SVR rates.^32^

Then came the Fusion trial. This assessed the efficacy of 12 weeks *vs* 16 weeks of therapy with SOF (400 mg OD) + RBV (1000 mg/day if < 75 kg or 1200 mg/day if > 75 g) in TE HCV-2 and 3 patients. Medication was given for 12 weeks, and then placebo for 4 weeks, in the first group, whereas the total treatment time was 16 weeks in the second group. At week 4, the SVR rates were 97 and 98% respectively. At the end of the pertinent treatment period, both groups had achieved SVR of 100%. However, on follow-up at 12 weeks, SVR was only 50% in the first group of patients, while it was 73% in the latter group. For genotype 3 specifically, the SVR12 was 30% in the 12-week group, whereas it was 62% in the 16-week group. Further subdivision of HCV-3 patients showed that, for cirrhotics, SVR12 was 19% in the 12-week group, but 61% in the 16-week group. However, in non-cirrhotics, SVR12 was higher at 12 weeks, 37%, but similar to cirrhotics at 16 weeks, 63%. This proved an additional benefit of extending the treatment duration to 16 weeks.^33^

The Positron trial was undertaken to assess the effect of SOF (400 mg once daily) + RBV (1000 mg/day if < 75 g or 1200 mg/day if > 75 kg) *vs* placebo in HCV-2 and 3 patients in whom pegIFN was not an option (IFN-intolerant, unwilling, or ineligible). In both groups, patients were treated for 12 weeks. Sustained virologic response rates were 99% at week 4, 100% at week 12 (end of therapy), and 78% 12 weeks after the therapy ended. No SVR was achieved in the placebo arm. In genotype 3 patients, there was 61% SVR12. Further subdividing based on cirrhosis showed 68% SVR12 in non-cirrhotic HCV-3 patients *vs* 21% in cirrhotic HCV-3 patients. The study concluded that SOF + RBV was an effective option in this group, but that it was more effective in non-cirrhotics.^33^

### SOFOSBUVIR, DACLATASVIR, AND ALISPORIVIR

Daclatasvir (DCV) is a new, oral, highly selective NS5A inhibitor, with broad HCV coverage *in vitro*.^34^ Earlier this year, Sulkowski et al studied the combination of DCV + SOF in treating chronic hepatitis C. Their study in genotype 2 and 3 patients was divided into three arms for previously untreated patients. Arm 1, consisting of SOF for 7 days, then DCV + SOF for 23 weeks, showed an 83% SVR at the end of treatment. Arm 2, SOF + DCV for 24 weeks, had the most astounding results, with a 100% response at week 24. Finally, arm 3 had DCV + SOF with the addition of RBV for 24 weeks, and also showed a good response rate of 93% at week 24.^35^ Similarly, the recent ALLY-3 phase III study assessed the use of the RBV-free combination DCV 60 mg + SOF 400 mg QD for 12 weeks, in patients with chronic HCV-3. It showed SVR4 rates of 91% in TN and 86% in TE groups. Out of a total of 152 patients, only 15 had relapse posttreatment, 11 of which were cirrhotic. The combination was well tolerated. The results supported the use of 12 weeks of therapy, rather than the expected 24 weeks, in non-cirrhotic patients.^36^ These results showed promise for DCV as a very efficient future treatment option. Alisporivir is another new drug that inhibits cyclophilin-A–NS5A interactions, thus regulating many phases of the HCV replication cycle.^37^ Garcia-Rivera studied the effect of Alisporivir in combination with NS5B inhibitors (SOF and Mercitabine) and NS5A inhibitors (DCV), and found it to have greater synergistic effects in HCV-3.^38,39^

### SOFOSBUVIR + GS-5816

Another promising NS5A + NS5B inhibitor combination, which has shown SVR4 rates of 100% when treated for 12 weeks, was the new regimen of SOF 400 mg + GS-5816 100 mg once daily. It was well tolerated and showed pan-genotypic coverage on initial study.^40^ Multiple phase 2 studies have shown high SVR12 rates of this combination after 12 weeks of treatment, with a low occurrence of adverse events.^41,42^ These trials are now studying its effect after 8 weeks of treatment, and are still showing 100% SVR4 and SVR12 rates according to Gane et al.^41^ However, further testing must be done to uncover its true potential of activity and adverse effects.

### DRUG–DRUG INTERACTIONS AND SIDE EFFECTS

Sofosbuvir is acted on by the P-glycoprotein complex and the breast cancer resistance protein (BCRP). Due to this, it is expected to be induced and inhibited by drugs acting on the P-glycoprotein complex. Inducers of this complex, which would potentially decrease SOF concentrations, include carbamazepine, phenobarbital, phenytoin, rifampicin, modafinil, and St John’s wort.^24^ Co-administration of SOF and the anti-retrovirals ritonavir/tipranivir is also not recommended as it decreases SOF concentrations as well.^23^ No interaction was seen between SOF and ethinyl estradiol/norgestimate,^43^ methadone,^23,24^ the NS5A inhibitors ledipasvir or GS-5816,^44^ or the immunosuppressants cyclosporine or tacrolimus.^45^

Sofosbuvir monotherapy has been seen to cause moderate anemia and mild degrees of headaches, fatigue, insomnia, nausea, rashes, dizziness, irritability, myalgia, and back pain.^46^ However, as mentioned above, very few treatment discontinuations were seen with it.

### RESISTANCE

Sofosbuvir has demonstrated a high genetic barrier to resistance.^47^ The primary mutation seen in the lab is the S282T mutation. However, clinically this was seen only in one genotype 2b patient in the Electron trial, receiving SOF monotherapy.^46^ It was not found in any patient in the above-mentioned trials who were receiving SOF in combination with RBV, with or without pegIFN alpha. New mutations seen in genotype 3a patients included the L159F and V321A variants. However, none of these mutations were associated with decreased susceptibility to treatment with SOF thereafter.^23^ Therefore, it can be concluded that relapse after therapy is not due to SOF resistance.

Concerning DCV, Sulkowski et al’s study demonstrated resistance polymorphisms in 5 of 18 genotype 3 patients. Pretreatment polymorphisms known to have caused loss of susceptibility to DCV *in vitro*, such as NS5A-A30K, were isolated. However, four of these five patients had a sustained virologic response.^35^

Alisporivir has been seen to have a high barrier to resistance. Viral breakthrough was seen only in patients with non-CC IL-28B allele and was associated with low drug dosage.^37^

In patients treated with SOF + GS-5816, Doehle et al showed that despite the presence of preexisting NS5A resistance associated variants (RAV’s) in 12 out of 54 HCV-3 cases, 9 did achieve SVR. These included the A30K and Y93H variants. The study concluded that despite the existence of these pretreatment variants, there was still a very high response to 12 weeks of treatment with GS-5816.^48^

### PERI-TRANSPLANTATION PERIOD

The use of SOF (400 mg OD) and RBV (weight based; 1000 mg/day if < 75 kg, 1200 mg/day if > 75 kg) in the pretransplant period, for 24 to 48 weeks or until the time of transplant, was proven by Curry et al to prevent HCV recurrence post-transplantation in 70% of patients who had undetectable HCV-RNA prior to transplant.^49^ This, in comparison to the previous response rate of 29% in HCV-3 patients treated with pegIFN and RBV pretransplantation,^50^ was a major milestone. For post-transplantation therapy, the AASLD recommends the use of SOF 400 mg + RBV starting at 600 mg, and bringing the dose up to the weight-based regimen slowly, for 24 weeks, in compensated HCV-3 cases.^27^

### SOFOSBUVIR IN SPECIAL CIRCUMSTANCES

#### HIV/HCV Co-infection

HIV co-infection is known to cause faster progression to fibrosis and a poorer long-term outcome.^51^ Sofosbuvir is the first DAA (direct-acting antiviral) to be FDA approved for use in HIV/HCV co-infection.^23^ The use of SOF + RBV for 24 weeks in this population showed 67% SVR for HCV-3.^52^ The AASLD recommends the use of SOF 400 mg OD + RBV weight based (same as for HCV mono-infected patients) for 24 weeks, in HCV-3/HIV co-infected patients.^27^

### RENAL FAILURE

Use of SOF and other DAA-based regimens becomes an issue when the GFR falls below 50 mL/min.^28^ The AASLD says that no dosage changes are required if GFR > 30 mL/min, but does not recommend SOF use if GFR < 30 mL/min, or if the patient is on hemodialysis.^27^ However, guidelines have not yet been established for an ideal alternative therapy in this population.

### HEPATIC IMPAIRMENT

In patients with mild-to-moderate hepatic impairment, raised SOF levels were seen. However, cirrhosis did not significantly alter its pharmacokinetics.^53^ No dosage adjustment is recommended in mild, moderate, or severe hepatic impairment. Concerning SOF in decompensated cirrhosis, guidelines have not yet been established.^23,24^

### CURRENT COSTS AND DRUG AVAILABILLITY

Currently, SOF, produced by Gilead as Sovaldi®, has been marketed at a price of $1,000 per pill or $84,000 for the whole 12-week course of medication, and costs are further increased by the need for drug combinations. This has made the drug inaccessible for thousands of people suffering from HCV worldwide, especially in countries with poor health care systems in place and where patients have to pay the whole cost from their own pockets. This has been a very distressing issue for both the physician and the patient population globally. Many civil society organizations and agencies have advocated that the price of this new medication be reduced to $500 per course, but as of yet, this has not happened.^54^ The WHO recently issued a statement urging price reduction for new medications like SOF, so that they can be used on a large scale.^55^

Egypt, at 14% HCV-affected population, has the highest prevalence in the world^56^ and has made headway in securing the whole course of SOF at $900 for a 12-week course.^57^ In France, the price of the 12-week course has been brought down to 41,000 euros ($51,373), thanks to the Economic Committee for Health Products(CEPS).^58^ In September 2014, Gilead has signed a deal with seven Indian pharmaceutical companies for them to develop SOF, and the single-tablet ledipasvir/SOF combination, for distribution in 91 developing countries (Table 1)^59^ at $10 per pill or $900 for the 12-week course^60^ (Table 2 gives a comparison of HCV-3 drug costs). Asian countries included in this deal are Pakistan, India, Afghanistan, Bangladesh, Bhutan, Cambodia, Indonesia, Laos, Kyrgyz Republic, Maldives, Mongolia, Myanmar, Nepal, North Korea, Sri Lanka, Tajikistan, Turkmenistan, Uzbekistan, and Vietnam.^59^ For the first time, patients in these countries will have a chance to fight HCV with these new, highly effective drugs. Awareness needs to be raised about the availability, and now cost-effectiveness, of this treatment option, as it could have a considerable impact on the global disease burden and economy.^61,62^

**Table 1:** **Table 1:** Countries included in Gilead’s generic manufacturing and distribution deal^59^

Afghanistan		Chad		Guatemala		Maldives		Rwanda		Swaziland	
Angola		Comoros		Guinea		Mali		Samoa		Tajikistan	
Antigua and		Congo, DR		Guinea-Bissau		Mauritania		Sao Tome & Pr		Tanzania	
Barbuda		Congo, Rep		Guyana		Mauritius		Senegal		Timor Leste	
Bangladesh		Cote d’Ivoire		Haiti		Mongolia		Seychelles		Togo	
Benin		Cuba		Honduras		Mozambique		Sierra Leone		Tonga	
Bhutan		Djibouti		India		Myanmar		Solomon Islands		Turkmenistan	
Bolivia		Dominica		Indonesia		Namibia		Somalia		Tuvalu	
Botswana		Egypt		Kenya		Nauru		South Africa		Uganda	
Burkina Faso		Equatorial		Kiribati		Nepal		South Sudan		Uzbekistan	
Burundi		Guinea		Kyrgyz Republic		Nicaragua		Sri Lanka		Vanuatu	
Cambodia		Eritrea		Lao PDR		Niger		St Vincent and		Vietnam	
Cameroon		Ethiopia		Lesotho		Nigeria		the		Zambia	
Cape Verde		Fiji		Liberia		North Korea		Grenadines		Zimbabwe	
Central Africa		Gabon		Madagascar		Pakistan		Sudan			
		Gambia		Malawi		Palau		Suriname			
		Ghana				Papua New					
						Guinea					

**Table 2:** **Table 2:** Costs of current HCV-3 drugs^60-62^

*Drug*		*Dose*		*12 weeks*		*24 weeks*		*48 weeks*	
PegIFN alpha-2a		180 μg SC 1 × /week		$6,000.00		$12,000.00		$24,000.00	
Ribavirin (RBV)		1200 mg daily		$3,000.00		$6,000.00		$12,000.00	
Sofosbuvir (SOF)-USA		400 mg daily		$84,000.00		$168,000.00			
Sofosbuvir (SOF) in developing countries included in the above deal		400 mg daily		$900.00		$1,800.00			

Daclatasvir, produced by Bristol-Meyers-Squibb as Daclinza®, has been approved by the European Commission for use in HCV-infected patients.^63^ It should soon be on the international market and its use should be incorporated into novel treatment regimens in Asia once that happens. GS-5816 is still in trials, and physicians and practitioners should keep this drug in mind as a highly effective therapy as well.^40,41^

### FUTURE NEEDS

Focus on future therapeutic development needs to be on an even lower occurrence of adverse effects, improved treatment efficacy in TE and cirrhotic patients, and use of drugs with a high barrier to resistance.

Of note, Simeprevir and the “Abbvie” regimen have not yet been proven to be effective in genotype 3 HCV-affected patients. More studies need to be undertaken with these drugs in order to establish a conclusive benefit, harm, or non-effect in HCV-3-affected patients.^39,64,65^
